# Behavioral and Dietary Habits That Could Influence Both COVID-19 and Non-Communicable Civilization Disease Prevention—What Have We Learned Up to Now?

**DOI:** 10.3390/medicina58111686

**Published:** 2022-11-21

**Authors:** Milica Veljković, Dragana R. Pavlović, Nikola M. Stojanović, Tanja Džopalić, Lidija Popović Dragonjić

**Affiliations:** 1 Department of Physiology, Medical Faculty, University of Niš, 18000 Niš, Serbia; 2 Department of Pharmacy, Medical Faculty, University of Niš, 18000 Niš, Serbia; 3 Department of Immunology, Medical Faculty, University of Niš, 18000 Niš, Serbia; 4 Department of Infectious Diseases and Epidemiology, Medical Faculty, University of Niš, 18000 Niš, Serbia; 5 Clinic for Infectology, University Clinical Center Niš, 18000 Niš, Serbia

**Keywords:** anti-inflammatory, antioxidant, COVID-19, immunity, prevention, supplementation

## Abstract

The massive expansion of the new coronavirus SARS-CoV-2 has urged countries to introduce lockdowns and set restrictive actions worldwide. The focus of the studies was to determine how COVID-19 induces damage to the lungs in order to find an alternative or adjuvant therapy that could lead to preventing COVID-19 or at least ameliorating it. This paper aims to survey the literature and provide new insights into behavioral and dietary habits that could influence the prevention of COVID-19. Maintaining an adequate mental health status, sleep, and taking moderate exercise are often disrupted in the conditions of lockdown and are followed by weakened immunity. Mediterranean and vegetarian diets are superior to other eating patterns in terms of immunity boosting and fighting COVID-19. Our study showed how adequate hydration, green tea intake, and supplementation with vitamins D, C, and E can increase our chances of avoiding the infection and even help us sleep better. Another focus of the research was on determining what level of hygiene really increases one’s chances of not contracting SARS-CoV-2, but this seems a little counter-intuitive at first. Since an immunocompromised state is a familiar predisposing factor for all contagious diseases, maintaining healthy behavioral and dietary habits could be a crucial step in boosting immunity and preventing COVID-19.

## 1. Introduction

In December 2019, the world was affected by the pandemic, which began with bouts of acute atypical respiratory disease in the city of Wuhan in the Hubei province of China. This rapidly spread from Wuhan to other areas, resulting in a human tragedy and tremendous economic damage. It was soon discovered that the culprit was a new coronavirus, a positive-sense single-stranded RNA virus [1,2]. It was identified as acute respiratory syndrome coronavirus 2, or SARS-CoV-2, and later renamed Coronavirus Disease-19 or COVID-19 [3].

At the beginning, it was thought that the onset of SARS-CoV-2 appeared via a zoonotic spread linked to the seafood market in Wuhan, China. It was then acknowledged that human-to-human dissemination was the real way the outbreak occurred [4]. COVID-19 was then determined to be a pandemic by the World Health Organization (WHO). Because of the rapid spread of COVID-19, countries worldwide embraced various restrictive measures aimed at stopping its transmission, including wearing masks, frequent hand washing, and social distancing [5]. With regard to social distancing, businesses, schools, community centers, and non-governmental organizations were forced to shut down, public meetings were banned, and restrictive measures were introduced worldwide, allowing travel for essential purposes only. The aim of all these restrictive measures was to enable countries to “flatten the curve” and avoid the collapse of their health-care systems [4].

SARS-CoV-2 virus symptoms can range from mild symptoms to critical respiratory insufficiency, with multiple organ dysfunction. The mean incubation period is 5.2 days. On a computerized tomography (CT) scan, the typical pulmonary ground-glass opacification can be found, even in patients who have no symptoms [6]. Since the virus predominantly attacks the respiratory system, it can cause fever, dry cough, sore throat, runny nose, and difficulty breathing [7]. Other organ systems can also be affected, causing symptoms, such as headache, dizziness, generalized weakness, vomiting, and diarrhea [8]. COVID-19-induced respiratory symptoms can vary greatly, extending from mild symptoms to severe shortness of breath and acute respiratory failure. In a report from Wuhan, the time between the appearance of symptoms and the development of acute respiratory insufficiency was only 9 days, suggesting that the respiratory symptoms may develop quickly and be severe. In addition, this virus could also be fatal [7].

Epidemiological data have demonstrated that mortalities were increased among the elderly [9] and that incidence was decreased among children [3]. Knittel and Ozaltun [10] documented a positive correlation between the number of the elderly, the incidence of commuting using community transport, and the frequency of SARS-Cov-2-induced deaths in the US. On the other hand, the authors provided evidence that obesity rates, intensive-care-unit beds per capita, and poverty rates were not linked to the lethality rate. The ongoing therapeutic solution is mainly supportive, with no identified and definite treatment obtainable yet.

## 2. Structure of SARS-CoV-2: Mechanism of SARS-CoV-2 Invasion into Host Cells

Coronaviruses are largely divided into four genera: α, β, γ, and δ, all based on their genomic structure, where α and β coronaviruses only infect mammals. At the moment, SARS-CoV-2 is classified as a β coronavirus [11].

The life cycle of the virus inside the host involves the following five steps: attachment, penetration, biosynthesis, maturation, and release. When viruses attach to host receptors they penetrate into host cells by fusing with their membrane through endocytosis. When viral RNA is incorporated into the nucleus, the process of replication begins and the virus makes its own proteins and releases its particles outside of the cell. Coronaviruses have four structural proteins: spike (S), membrane (M), envelope (E), and nucleocapsid (N) [12]. It has been found that SARS-CoV-2 has a functional and attachment affinity towards receptor ACE2, whose expression is high on the apical side of lung epithelial cells. The spike protein binds to ACE2 and after that undergoes a protease cleavage [13]. In order to establish whether or not it has another receptor it can bind to, further research needs to be conducted.

## 3. Host Response to SARS-CoV-2

Since ACE2 is highly expressed on the apical side of lung epithelial cells in the alveolar space [14], this virus is likely to penetrate and damage them, even causing apoptosis. T-cell responses induced by COVID-19 are initiated by antigen presentation via dendritic cells and macrophages (which belong to antigen-presenting cells—APCs). These APCs can either phagocytize apoptotic cells infected by the virus [15] or are infected by the virus on their own. This question remains unclear, because ACE2 receptors are barely present on dendritic cells and macrophages, so further studies are needed to find out if SARS-CoV-2 uses another receptor to bind to cells.

Immunological research was mainly conducted on adult COVID-19 patients with critical disease, showing lymphopenia and depletion of T lymphocytes [9,16]. These patients were found out to have elevated levels of pro-inflammatory cytokines, including IL-6, IL-10, GM-CSF, MCP1, MIP1α, and TNF-α [7,9,16]. The more critical the state patients were in, the more increased their serum IL-6 levels were. Depletion of T lymphocytes could be the reason why their disease advanced. Another discovery was that abnormal pathogenic CD4+T lymphocytes expressing both IFN-γ and GM-CSF were found in COVID-19 patients with harsh illness [9]. Although GM-CSF was found to help innate immune cells to develop into T cells, it can also be responsible for massive tissue destruction [17].

Research shows that respiratory cells attacked by coronavirus generate IL-8 (as well as IL-6) [18], which acts as a chemoattractant for neutrophils and T lymphocytes. Severe COVID-19 patients are found to have massive inflammatory infiltrates in their lungs where the most dominant cells from innate immune response are neutrophils. These same neutrophils are the ones that induce tissue destruction [19]. The most dominant cells in the infiltrate that belong to the adaptive immune response were cytotoxic CD8+T cells, which can explain the depletion of T-cell count in peripheral blood. These T cells can kill the virus, but similarly to neutrophils, they can also cause tissue destruction [20]. Other cells found in the infiltrate were monocytes, which respond to GM-CSF and have high expression of IL-6. It is very probable that their number rises with the progression of the disease.

In addition to respiratory symptoms, severe COVID-19 patients suffer from thrombosis and pulmonary embolism and have elevated levels of fibrinogen and d-dimer. This hypercoagulable state most likely implies massive endothelial damage. Since endothelial cells comprise one-third of pulmonary cells [21] and they express ACE2 [22], the virus could penetrate them, causing their injury. Endothelial injury could cause increased vascular permeability, which can make viral penetration easier.

## 4. Aim

The goal of this paper is to survey the existing literature on the onset of COVID-19, its outbreak and mechanism of action in the infected organism, as well as the habits in behavior and diet that could prevent the disease or at least ameliorate it. This paper is also meant to present new insights from numerous published results.

## 5. COVID-19 Impact on Individual’s Physical Health and Ways to Improve It

Chatterji and Li [23] studied the effect of COVID-19 on the US health-care sector. They discovered that the pandemic was linked with a 67% drop in the total number of outpatient visits per provider by the week of 12–18th April 2020 compared to the same week in the previous years. This might have negative health consequences, especially amongst individuals with chronic illnesses. Others, such as Alé-Chilet et al. [24], documented the decline in emergency visits to hospitals worldwide. Since people seldom undergo medical checkups during pandemics, overall health could be maintained and taken care of by adopting positive habits in everyday behavior and diet, which is what we are going to cover in this paper. These same habits could, in addition, lead to the prevention of COVID-19 or at least amelioration of the disease.

## 6. Mental Health in COVID-19 Pandemics

Governments across the globe imposed “lockdowns”, which confined many people to their homes. This interferes with normal life routines, which are important for maintaining circadian rhythm. The pandemic is also associated with new stressors, altered roles, and uncertainties about health and economic security, which are likely to affect both mental health status and sleep [25,26,27]. What accompanied the lockdown was a drop in in-person social contact, which elevated the rate of loneliness. Increased loneliness, on its own, may lead to an increase in mood disorders, self-harm, suicide, and worsen the pre-existing emotional state [28]. Loneliness is also linked to the deterioration of physical health and increase in mortality risks [29]. Without strategic help, continuous loneliness might have a paramount adverse influence on health and well-being [30]. People who have jobs with huge responsibilities during the COVID-19 outbreak, especially those in the medical profession, are under enormous pressure, and there is a risk that their emotional well-being may be jeopardized [28]. However, a study conducted during the SARS pandemic discovered that loneliness was documented in both medical workers and non-medical workers [31]. Relative to the pandemic, the rate of loneliness was increased for those in quarantine, either because they are thought to be high risk or have been instructed to put themselves in quarantine. However, loneliness was not associated with being in or having been in lockdown. In addition, the chances of experiencing loneliness were not increased for crucial employees or for the population who took care of someone with COVID-19 [30].

The American Medical Association documented impairment in mental health status and well-being for a large part of the population, manifesting as feelings of social isolation, distress, anxiety, boredom, frustration, loneliness, and depression [30]. Béland et al. [32] found that those most prone to lower mental health status were the elderly and employed populations, whose levels of education were below high school. Their study also showed that those who missed work or were unemployed experienced impairment in mental health. In addition to the elderly and those who were not regularly at the work place, another part of the population that was very susceptible to a deterioration in mental health was women due to increased parenting responsibilities [33]. Armbruster and Klotzbücher [34] documented that there was an increase in the need for psychological help (through SOS calls) owing to the quarantine restrictions established in Germany. The authors discovered that the main reason for these calls was depression and loneliness. In Serbia, a clinical survey revealed that there were significantly more suicidal patients during 2020 than in the previous years, which was found to be related to the constant exposure to COVID-19 infection information from the media [35]. The issue related to the deterioration of mental well-being in times of the COVID-19 pandemic is that psychological stress, on its own, damages immune response by increasing pro-inflammatory cytokines, such as IL-6 [36]. This cytokine is also elevated in COVID-19 infection, inducing lung damage, so a decline in mental health status could potentially make an individual more prone to COVID-19 or worsen the progression of the illness. In order to hopefully prevent COVID-19, it would be wise to take care of mental health and reduce stress, which can be done via various mindfulness techniques, such as meditation, breathing exercises, or guided imagery. When applied correctly and regularly, these methods are documented to relax, reduce tension, reduce activated NF-kB, reduce CRP, and prevent inflammatory cytokines from increasing [37]. When the demand for more psychological help is present, it is recommended to attend an online therapy session in the comfort of one’s own home to avoid the possibility of an infection during the pandemic.

## 7. Regular and Adequate Sleep

Sleep has a huge impact on the immune system and gives the body a chance to heal and rest, especially in critical diseases. In addition, sleep was considered essential by physicians in the recovery of their patients during the Spanish Flu Pandemic [38]. Gupta et al. [39] conducted an online survey on sleep experience, routines, physical activity, and symptoms of anxiety and depression, to study the alterations associated with the lockdown. In comparison to the pre-lockdown period, there was a shift to a later bedtime and waking time, with a drop in night sleep and an increase in sleeping during the day. Sleep quality was impaired among groups. A decrease in sleep length was linked to depressive mood. About a quarter of people from this study (23.4%) were documented to have lower sleep quality in comparison to the pre-lockdown state. Another study, in the Greek population, discovered that almost 38% of the study subjects had clinical insomnia following COVID-19 onset [40]. The participants involved in this research were younger in comparison to the ones involved in the research of Gupta et al. [39].

Daytime napping, reported by people during lockdown, reduces sleep pressure and, hence, postpones the bedtime and extends sleep-onset latency [25,41]. In addition, it was documented that postponed sleep shortens the part of slow-wave sleep due to circadian elements and this might elicit bad sleep efficiency as well [25,41]. Additional elements that might impact sleep patterns could be screen time, which was extended following lockdown. Increased screen time is linked to decreased sleep duration and poor sleep quality [42].

The pandemic lockdown is linked to the alterations in sleeping habits and in the efficiency and amount of night-time sleep. In spite of these alterations being linked to a drop in mental health status, it is indistinct from the current data whether sleep impairment induces psychological pain or vice versa.

A solution to this problem could potentially be vitamin D supplementation. Vitamin D has an essential role in adjusting sleep patterns, circadian rhythm, augmenting sleep efficiency, and secondarily relieving sleep apnea [43]. People with clinical insomnia are associated with vitamin D deficiency [44]. In addition to having beneficial effects in regulating sleep, vitamin D could potentially have many other positive effects in preventing COVID-19 that we are going to cover in this paper.

## 8. Exercise as a Source to Prevent COVID-19

Numerous positive effects of exercise are known. In this paper, we will focus on the ones that are important for fighting COVID-19. Exercising induces the number of leukocytes and antibodies to increase protection against infections [45]. Exercise is particularly crucial after a critical disease to recover muscle mass, strength, and resiliency [45,46]. Exercise can also play a role in inhibiting the formation of blood clots, which COVID-19-infected people are prone to [47].

Moderate exercise with less than 60% of maximal oxygen uptake (VO_2_max) has a positive influence on both innate and adaptive immune cells. It especially augments the activity of natural killer cells, neutrophils, and macrophages [48]. Acute aerobic exercise (i.e., aerobics or spinning class) has been documented to elevate monocyte count [49], which has plays an essential role in fighting viruses. Exercise causes monocytes to differentiate within 18 h into mature dendritic cells, with augmented ability to activate T lymphocytes [50]. Prolonged moderate exercise training is especially beneficial to high-risk groups for COVID-19 infection, such as the elderly, postmenopausal women, immunocompromised, and cancer reconvalescents, since it increases T-cell proliferation and the number of T-helper cells [51].

On the other hand, severe-intensity exercise can be counter-productive when it comes to a tendency toward infections because it is related to the higher frequency of upper-respiratory-tract infections among world-class athletes [52]. The concentration of secretory antibodies that line and protect mucosal surfaces, IgA and IgM, decreases right after high-intensity training, but ordinarily recuperates within 24 h [52]. This kind of exercise elicits temporary oxidative stress and disruption of muscles, cellular integrity, and homeostasis, particularly in the course of the first 24 h after exercise [53,54]. It also augments recombinant IL-2 stimulation of NK cells right after acute intense training [55]. Acute repeated high-intensity trainings have been documented to cause displacement and regeneration of aged immune cells, particularly cell senescent naïve, memory CD4+ and CD8+ T-lymphocytes, and an increased number of apoptotic lymphocytes in peripheral blood [56]. The way exercise imposes cardiovascular, respiratory, and metabolic changes, which result in higher maximal oxygen uptake in the body, is through transient stress (i.e., disruption of cell homeostasis and oxidative stress) elicited by an acute exercise bout. As a result, higher maximal oxygen uptake can be responsible for long-lasting improved immune response, particularly when exercise is performed constantly [48].

The immune system is said to be better in people active in sports versus sedentary individuals. Repercussions of sedentary lifestyle and lack of physical activity include an immunocompromised state due to systemic inflammation, oxidative stress, and linked immunosuppressive processes [57]. Superior physical condition and fitness levels in adults are linked to an immunological boost elicited by decreased C- CRP, IL-6, IL-18, TNF-α, and other inflammatory indicators [58]. Hence, any kind of exercise is advantageous in comparison to sedentary lifestyle, particularly throughout the COVID-19 pandemic. Moderate exercise intensity is particularly recommended since it has the most positive effects, which include reaching a rise in VO_2_ max as a pragmatic goal, especially for individuals at high risk of infection [59]. It is especially welcome to perform moderate exercise at home in order to avoid the airborne coronavirus and boost one’s immunity in order to prevent it at the same time [60,61]. It is very important that the exercise performed during the pandemic is moderate in intensity and that high-intensity training is avoided. Although high-intensity interval training (HIIT) has been documented to adjust systemic inflammatory reactions, involving decreasing plasma ALT and AST levels, there is no evidence that it brings more welfare than regular moderate exercise. On the contrary, high-to-peak exercise intensities (such as high-intensity training—HIT) can induce systemic inflammation response, oxidative stress, and muscle damage, hence, deranging the immune response [62]. A concrete way would be to organize training in a way that uses 40–70% of maximum heart rate. Regular exercise training with moderate intensity exhibits superior anti-inflammatory features to short bouts of non-regular workouts [62]. Recommended types of exercises are: strengthening the core muscles, stretching, or a combination of these (hiking, raising and holding moderate weights by using house items, climbing stairs, yoga, or aerobic exercises following an online instructor). A weekly volume increase should be from 150 min to 200–400 min aerobic exercise, distributed across 5–7 days, with at least 2–3 resistance sessions with a progressive increase in weight lifting (only 10% per week). It is particularly welcome that exercise becomes part of a multipart individual lifestyle outlook (individualized diet, workout strength, technology, behavior, mental health status) [63].

## 9. Hygiene in COVID-19 Pandemic

During the COVID-19 pandemic, the extensive use of hand sanitizers and frequent hand washing are especially encouraged. However, in some developed countries, the reported fatality rate is far higher than in others. Further, the WHO documented that patients infected in low-socioeconomic conditions have low rates of a severe and lethal infection compared to those raised in more hygienic circumstances. Researchers argue that the hygiene hypothesis [64] may apply to COVID-19 susceptibility and that the population in low-hygienic conditions acts, so as to train innate immune defenses to minimize the severity of infection. The hypothesis supported the view that children who had contact with various allergens, such as farm animals, home pets, and enteric parasites, were less prone to developing allergies and some autoimmune conditions than those who had a more hygienic childhood [64]. Certainly, at least in some Western countries, long before COVID-19, large utilization of hand cleaners and constant hand washing were encouraged when raising children. To be more precise, people who live in lower-hygienic conditions, according to the hygiene hypothesis, train their innate immune response to be more robust. Therefore, to our surprise, they seem to be less susceptible to COVID-19 infection and even if they get it, it appears to be milder than in the population who lives in developed countries with better hygiene conditions [65]. However, despite these controversial but interesting data, regular hand washing, hygiene, and wearing masks are extremely important during this pandemic and should not be neglected.

## 10. Dietary Habits That Could Help in Prevention of COVID-19

Disparities in nutrition may result in a suboptimal diet that is associated with several cardio-metabolic conditions linked to COVID-19 infection and its prognosis [66,67,68]. Being on an unhealthy diet causes inflammation and oxidative stress in an organism, which can, on its own, adversely affect homeostasis and immune response. Hence, it would be essential to adopt healthy dietary habits during the pandemic and maintain them, as advised by the WHO. According to our online survey [69], the age of the examinees and their previous dietary habits greatly influence the frequency of consuming fruits, vegetables, herbal teas, and supplements during the pandemic. Elderly survey participants showed greater discipline in eating plant functional food, probably because they belong to a vulnerable group when it comes to this infection. A notable shift towards higher utilization of herbal teas and dietary supplements could be seen, particularly among the people that used them before from time to time.

Optimized nutritional patterns enhance the immune response through alterations in signaling molecules, enabling cellular activation and gene expression. Regarding this, different nutrients constitute gut microbioma and make immune reactions in the organism adequate. Therefore, various investigations document that boosting the immune response represents one supportable method to enhance the likelihood to make it through in these pandemic circumstances [70].

Since we have already explained how COVID-19-induced inflammation caused damage to the organism, in the next part of the paper, we will cover what kind of foods and supplements would be beneficial to introduce into the everyday diet in order to prevent the infection or at least make it milder and avoid the severe kind of disease.

## 11. Hydration

People at risk of COVID-19 infection must drink plenty of water because it will keep their mucous membranes moist, which helps to decrease the risk of being infected in the first place. Other fluids are also appreciated and can help, such as coconut water, soups or broths, milk, green tea, or freshly squeezed fruit juice. If the liquid a person drinks is a source rich in antioxidants, it becomes even more efficient in fighting the infection. While drinking water does not guarantee avoiding the infection, remaining hydrated benefits one’s health and ensures that the immune response remains strong enough to fight the infection if it comes to that. Water drinking assures that cells gain enough oxygen. That way, they stay vital and can combat the infection more vigorously. According to the Centre for Disease Control and Prevention, hydration often plays an essential role in keeping track of body temperature. Nevertheless, if someone has a fever, as an outcome of COVID-19 disease, drinking enough water is of crucial importance in order for the organism to be able to drop it. Staying hydrated ensures that the nutrients taken from food are properly transferred everywhere through the body so that all physiological functions can be performed the way they are supposed to. This, again, increases the chances of fighting the infection. Sometimes, dehydration and dryness of mucous membranes can be induced by antiviral drugs. Further, when people are ill, they tend to dehydrate and lose plenty of fluids in the form of mucus in order to kill the infectious agent. As long as people drink enough water, they can get rid of both mucus and microbes all together. Even if staying hydrated does not prevent a person from contracting the notorious SARS-CoV-2 virus, it will surely help to recover from the disease [71].

## 12. Vitamin D

It is already well known that since the outbreak of COVID-19, vitamin D supplementation has been especially encouraged. Vitamin D reduces acute respiratory tract infection and its deficiency is linked to susceptibility to viral infections and also to various cancers, diabetes, and cardiovascular diseases [72,73,74,75]. It enhances differentiation of monocytes to macrophages and augments their fighting ability; mediates the generation of inflammatory cytokines; and supports antigen presentation. In addition, vitamin D metabolites appear to regulate the creation of specific antimicrobial proteins that directly kill microbes and, therefore, tend to assist in mitigating infections, including the respiratory ones [76]. Numerous researchers discuss how vitamin D lowers the risk of a viral infection and fatal outcome [77]. There are several pathways to do so. There is a study that investigates the capacity of vitamin D to reduce the likelihood of the common cold, where the activities of vitamin D are categorized into three groups: adaptive immunity, physical barrier, and natural cellular immunity. Vitamin D enhances gap junctions, tight junctions, and junctions of adherents (i.e., by E-cadherine). Various studies pointed out how viruses injure the integrity of the junction, growing virus contagion as well as additional pathogens. Vitamin D improves cellular innate immunity, partly by eliciting the production of antimicrobial peptides, such as human cathelicidin, LL-37, and defensins [78]. Studies report a link between decreased blood concentrations of 25-hydroxyvitamin D and susceptibility to acute respiratory-system infections [79]. Martineau et al. [73] concluded that daily or weekly intake of vitamin D defends from acute respiratory infections in general and it is considered to be safe. Vitamin D augments defense of the cells as well by increasing cytokine production in the innate immunological response. It is documented in COVID-19 patients that the innate immune system produces both anti-inflammatory and pro-inflammatory mediators as a reaction to microbial infections. Vitamin D is able to reduce the production of pro-inflammatory Th1 cytokines, TNF-α, and INF-γ. It can also increase the production of anti-inflammatory mediators by macrophages, thus, acting as an immuno-modulator [80].

Vitamin D concentrations progressively drop with ageing. This might be important for COVID-19 patients since the disease is more severe and more fatal with age. Reasons for decreasing vitamin D concentration with age might be shorter time spent in the sun or decreased generation of vitamin D in skin due to lower skin levels of 7-dehydrocholesterol. In addition, elderly people tend to use more drugs, including cytostatics, anticonvulsive, antihypertensive, anti-inflammatory drugs, or antibiotics, that stimulate the pregnane-X receptor and, thus, reduce blood 25-hydroxy cholecalciferol levels. While we can take in vitamin D through the diet; it is mostly produced internally by exposing the skin to UVB radiation. Actually, spending time in the sun could elevate blood vitamin D concentrations at the same rate as the intake of 250 to 625 μg of vitamin D orally. The Societa Italiana di Nutrizione Umana suggested exposure to sunlight for 15 to 30 min daily to enhance the production of vitamin D [81]. Since vitamin D is a powerful antioxidant, its intake reduces the use of another antioxidant, vitamin C, which fights infections as well [82].

As we already mentioned earlier in the paper, sleep disorders are frequent during pandemic-imposed lockdowns and can be managed through vitamin D supplementation [83]. It would be extremely beneficial since vitamin D has an essential part in regulating sleep patterns, circadian rhythm, improving sleep efficiency, and indirectly ameliorating sleep apnea [43]. Vitamin D receptors and the enzymes that manage their activity and break down are discovered in brain areas responsible for sleep adjustment. Vitamin D is also included in melatonin-generation pathways and has an impact on restless leg syndrome and obstructive sleep apnea syndrome [84].

## 13. Vitamin C

Vitamin C is an important aspect in improving immunity, for children, adults, or even elderly people. Water-soluble vitamins have important benefits in treating sepsis and septic shock, a critical condition, induced by inflammation [85,86]. Vitamin C protects the organism as a pro-oxidant for immune cells, antioxidant for lung epithelial cells, and fights immunosuppressive states [85,86]. Foods that contain vitamin C are oranges, bilberry, papaya, guava, kiwi, kale, beetroots, spinach, cauliflower, and broccoli [87]. Vitamin C impacts several aspects of immunity, including supporting epithelial barrier function, development and activity of both innate and adaptive immunity response, leukocyte chemotaxis towards inflammation, phagocytosis and ingestion of pathogens, and antibody generation [74]. Similar to flavonoids, vitamin C fights against NLRP3 inflammation activity [88]. Clinical trials documented that ascorbic acid decreases the recurrence, duration, and harshness of the common cold and pneumonia [89]. Functional foods, such as bilberry, which contain many other precious dietary components, in addition to vitamin C, could be beneficial for maintaining good health [90,91]. A usual daily dose of ascorbic acid varies from 500 mg to 3000 mg, with even higher doses used in periods of acute infection.

## 14. Vitamin E

Vitamin E is essential for maintaining the immune defense response in older populations. It is also a powerful antioxidant that can protect against infection, inflammation, and oxidative stress [92]. Foods rich in vitamin E are: almonds, peanut butter, sunflower seeds, and even hazelnuts. As a part of its antioxidative activity, vitamin E shields polyunsaturated fats in plasma membranes and lipoproteins [93]. Vitamin E deficiency has been linked with harming both humoral and cellular immunity. Its supplementation was found to have positive effects in some viral infections. When chronic hepatitis B patients were supplemented with vitamin E, a substantially higher normalization of liver enzymes and HBV-DNA negativization was documented in the vitamin E group [94,95]. It has been prescribed, together with vitamin D and C, as a treatment for prophylaxis of COVID-19 infection.

## 15. Selenium and Zinc

Micronutrients are vitamins and minerals that are needed in small amounts in order for homeostasis to be maintained. Their deficiency disturbs the equilibrium of an organism and weakens the immune response [96]. Rayman [97] documented that selenium (Se) insufficiency is linked to a lower immune response. Meanwhile, selenium supplementation has a stimulating effect on immune response, which includes augmenting of activated cytotoxic T cells and NK-cell activity [98]. COVID-19-induced disruptions in sensory cells in oral mucosa may be the reason for parageusia. Chelation of zinc (Zn) through immune mechanisms induces a change in zinc homeostasis in oral gustatory cells. As reported, this can lead to taste disorders caused by zinc deficiency [99]. Hence, both Se and Zn supplementation could have a crucial part in the prevention and therapy of COVID-19 [100].

## 16. Mediterranean Diet

An adequate and balanced diet represents an essential base for an optimal immune response. It helps to maintain the antibody generation and assures the consumption of nutrients that are able to modulate inflammation and oxidative stress [101]. Over the last 30 years, the high use of a high-fat diet has been proven to have a negative impact on immunity. Saturated fatty acids act on Toll-like receptor 4, a sensor that binds bacterial lipopolysaccharide and, thus, acts in the innate immune response. As a result, a high intake of saturated fatty acids favors low-grade inflammation. Conversely, polyunsaturated fatty acids (such as omega-3 in fish, which is highly present in the Mediterranean diet) are known for their favorable activity on both innate and adaptive immunity [81]. Mediterranean diet, which has represented the standard dietary pattern of people who live near the Mediterranean Sea since the 1960s, involving Greece and South Italy, has, for an extended period of time, been indicated as an eating habit capable to maintain heart and metabolism well-being. The Mediterranean diet is composed of an abundance of plant-based foods, olive oil as the main source of fat, low to moderate consumption of fish, dairy products, and poultry, low intake of red or processed meat, and low to moderate drinking of wine with meals [102]. Various epidemiological investigations verify the anti-inflammatory and immuno-modulatory effects of Mediterranean diet on various illnesses linked to chronic low-grade inflammation. These effects oppose the activities of various inflammatory mediators [102]. A recent meta-analysis compared individuals on the Mediterranean diet with those on a control diet. Consuming the Mediterranean diet for two years was connected with a marked drop in inflammatory mediators, involving CRP, IL-6, IL-7, and IL-18, in comparison to a prudent dietary pattern [103]. In 2017, Calatayud et al. [104] studied the favorable results of the Mediterranean eating pattern in patients with frequent colds and recurrent respiratory complications and found out that after one year, hospitalizations indicated a positive and statistically significant progression, implying that the Mediterranean diet could enhance the outcomes of children with repeating colds and frequent inflammatory complications in the respiratory tract.

However, there is a certain challenge concerning the compliance of people with the Mediterranean diet during lockdowns. This confinement to home, imposed by government, including the one in Italy, completely altered people’s lifestyle and behavioral habits. As a repercussion of the lockdown, the restricted approach to daily grocery purchase may have limited the intake of fruits, vegetables, and fish, in comparison to processed food. Meanwhile, the latest Italian research documented that throughout the quarantine, the Italian population stayed committed to the Mediterranean diet, sustaining a high nutritional quality, particularly in Northern and Central Italy, in which a decreased body mass index was documented in comparison to the regions of Southern Italy and the islands [105]. In addition, individuals that were able to comply with the diet ate more than 50% of olive oil, vegetables, such as wild garlic [106] and legumes, foods abundant in the Mediterranean dietary pattern, showing that being on a wholesome diet is manageable, even in emergency times. Thus, the Mediterranean diet could be a promising therapeutic and strategic way to target the susceptibility to COVID-19 infection, prevent the development of severe illness, and improve the mortality and overall well-being of the affected people [101].

## 17. Vegetarian Diet

It is implied that chronic gut inflammation results from a specific composition of gut microbiome regulated by food intake. Plant-based foods are likely to support a gut microbiome capable of inducing an appropriate level of anti-inflammatory immune response in the host, in contrast to a pro-inflammatory immune response elicited by the gut microbiome of individuals consuming foods, such as wheat, red meat, and alcohol, thereby resulting in chronic gut inflammation [106,107]. It is documented that vegetarians have 30% less mortality caused by respiratory diseases than people who consume meat [108]. The “Western” diet, habitual in developed countries, is defined by an increased intake of processed red meat. This dietary pattern is linked to an elevated risk of asthma in children [109], which is frequently treated with some types of honey products [110]. A possible way that red meat increases the risk for asthma is through preservatives it contains, such as nitrates, which extend its shelf life. Studies document that there was a rise in the number of bacteria that belong to *Firmicutes phylum* with a coinciding decline in the bacteria of *Bacteroidetes phylum* in the intestines of people who live in Western countries. Generally, *Bacteriodetes* are documented to be abundant in populations on vegetarian diets, whereas *Firmicutes* are abundant in populations on animal-based diets [111]. According to these data, when it comes to COVID-19 prevention, it seems that a vegetarian diet works more protectively because it stimulates growth of the better kind of bacteria in the digestive tract, which would enhance anti-inflammatory response. It also seems that the intake of synbiotics (prebiotics and probiotics) or foods that contain them would be beneficial in terms of preventing COVID-19. In the early 1990s, Elie Metchnikoff published that Bulgarians live very long, which was explained by the intake of yoghurt, thereby reaching the requirement of maintaining a good microbiota to sustain homeostasis [112]. Developed countries tend to be on animal-based diets and developing countries (i.e., India) tend to be on plant-based diets. Even immigration from a developing country to the US has been documented to westernize the eating pattern and human gut microbiome [113].

At the same time, a transfer from an animal-based diet to a plant-based diet, including a fiber-abundant diet, has been reported to be linked to an alteration in gut bacteria (that alter within days to weeks) [105]. Such a transfer was documented throughout the quarantine in the Indian population. This could have ameliorated any previous chronic inflammation, thus, reducing the development of cytokine storm. As a consequence of lockdown, it can be assumed that the population had a restricted approach to animal-based or pre-cooked refined food, which is principally abundant in refined sugars and fats. The quarantine very fortunately directed the population towards the intake of a domestic wholesome diet, which possibly caused a decrease in frequency/gravity of illness throughout this period because of an enhanced microbiota or symbiosis [114].

## 18. Green Tea

Green tea (*C. sinensis*) contains catechins or polyphenolic compounds, which are epigallocatechingallate (EGCG), epigallocatechin (EGC), epicatechingallate (ECG), epicatechin (EC), gallocatechin-3-gallate (GCG), gallocatechin (GC), catechingallate (CG), and catechin (C) [115,116]. These green tea catechins are documented to exhibit antiviral activities against many viruses. Epigallocatechingallate (EGCG) is one of the most abundant and powerful polyphenoliccatechins found in green tea. It is investigated for its antiviral action against various viruses and is documented to be a potential therapy solution over traditional medicine. It is also known as a healthy functional food, showing anti-cancerous, anti-inflammatory, antibacterial, antioxidative, and antiproliferative activities, in addition to its antiviral effects [115,117]. All the polyphenols exhibit adequate absorption in the human gut as well as good elimination (except CG and ECG) from the body. In addition, these catechins have a maximum tolerance dose (expressed in terms of log mg/kg/day), ranking from 0.438 to 0.506, which makes them safe for human use. This result implies that green tea polyphenols/catechins can be suitably utilized as therapy [118]. Recent data discovered that many active substances, including a polymerized polyphenol (Oolonghomobisflavan-A), from green tea act as effective SARS-CoV-2 main protease (Mpro or chymotrypsin-like protease) inhibitors [119]. Another study showed that another three polyphenols (EGCG, ECG, and GCG) interacted with the catalytic residue(s) of the Mpro protease and exhibited higher binding affinity than a well-known Mpro protease inhibitor (N3); it is safe to conclude that they could probably impede the catalytic activity of Mpro protease and be the successful therapeutical solution for the therapy of COVID-19 infection. Additionally, binding free energy evaluations using the MM-GBSA method also shows that Mpro–GCG complex (53.54 kcal/mol) is relatively more stable than Mpro–ECG (48.92 kcal/mol) and Mpro–EGCG complex (43.56 kcal/mol). In addition, this investigation reveals future possibilities for testing (in vitro and in vivo) these three green tea catechins in the treatment of COVID-19 [118]. A recent target-based virtual ligand screening study implies that at least one polyphenol (EGCG) is likely to attack papain-like proteinase (PLpro), an essential enzyme in the process of coronavirus replication and infection of the cell [120]. In addition to data that EGCG binds to Mpro, it is also documented to be beneficial in illnesses with unrestrained immune activation; therefore, it could ease the gravity of COVID-19 [121] since the latter refers to an exaggerated activation of the immune response [122]. Overall, these data imply that green tea polyphenols could decrease, for the most part, the danger related to COVID-19. Accordingly, it could be anticipated that regions with increased green tea intake are less afflicted by COVID-19. Indeed, Storozhuk [123] discovered that there are poor but statistically significant correlations among morbidity and mortality and green tea intake at the level of individual countries.

## 19. Conclusions

COVID-19 expanded at a disturbing rate and the absence of an accepted therapy created an enormous burden on health-care facilities. The current circumstances with the SARS-CoV-2 pandemic, its alarming consequences, and the yearly morbidity and mortality overall make it clear that public-health habits, such as hygiene measures, use of masks, social distancing protocol, and government-imposed lockdowns, alone are not sufficient. Thus, antiviral drug and vaccine development is essential to fight this pandemic situation. In addition, individual immune boosting is one more plan to battle this disease. Therefore, a distinct guidance is required in terms of which behavioral and dietary habits could positively serve as a means of preventing COVID-19 infection or at least ameliorating its severity if it failed to be avoided. Taking care of mental health, regular sleep, and moderate workout in conjunction with increased dietary intake of functional food in the ways described in the paper may contribute as a preventive therapy against the emergence of future pandemics. These habits could also be beneficial for ameliorating the severity of the disease and have an overall positive effect on immunity and health, even when the pandemic ends.

## Data Availability

Not applicable.
